# Supplementary material for: The needs and opportunities of older laypeople to acquire first aid skills
Source: PLoS One. 2021 Oct 12;16(10):e0255964. doi: 10.1371/journal.pone.0255964 (PMC8509921; doi:10.1371/journal.pone.0255964)
Supplement: S1 Appendix — (DOCX) [file pone.0255964.s001.docx]

**APPENDIX: FIRST AID QUESTIONNAIRE**

**SECTION KNOWLEDGE**

1. **Please, assess your first aid knowledge (either theoretical or practical) on a scale from 0 to 10 (0 - the lowest grade, 10 - the highest grade; 0 means you don't have any first aid knowledge, and 10 means you know first aid very well).**

| 0 | 1 | 2 | 3 | 4 | 5 | 6 | 7 | 8 | 9 | 10 | I do not know. |
| --- | --- | --- | --- | --- | --- | --- | --- | --- | --- | --- | --- |

2. **Which phone number would you call if you needed urgent medical assistance in Slovenia?**

| 112 |
| --- |
| 113 |
| Other |
| I do not know. |

3. **What do you think is the right ratio of chest compressions and rescue breaths at an adult?**

| 5 chest compressions on 1 rescue breath |
| --- |
| 15 chest compressions on 2 rescue breaths |
| 30 chest compressions on 2 rescue breaths |
| I do not know. |

4. **Please, answer the following statement: 'Yes' if you think the statement is correct, and 'No' if you think the statement is wrong.**

If anyone starts coughing during the eating (due to airway obstruction), we should encourage them to continue to cough.

| Yes |
| --- |
| No |
| I do not know. |

5. **Please, answer the following statements: 'Yes' if you think the statement is correct, and 'No' if you think the statement is wrong**.

|  | Yes | No | I do not know. |
| --- | --- | --- | --- |
| We stop severe bleeding by the direct pressure on the bleeding site. |  |  |  |
| The amputated part is not placed directly on ice. |  |  |  |
| In case of bone and joint injury, a person should not eat and drink. |  |  |  |
| After the fracture of a hip, the injured person should be taken to a health facility. |  |  |  |

**SECTION ATTITUDE TOWARDS FIRST AID**

**6. You have found yourself in a situation when someone in the street needs first aid. Would you be willing to give it, or would you rather leave it to others?**

| I would give first aid myself. |  |
| --- | --- |
| I would rather leave it to others. |  |
| I do not know. |  |

**7. Have you ever been on first aid training in the past or gained first aid knowledge in any other way?**

| Yes |
| --- |
| No |
| I do not know. |

**8. IF Q 7 IS YES - How many years have passed since your last first aid training/education?**

| 1 year or less |
| --- |
| Between 2 to 5 years |
| More than 5 to 10 years |
| More than 10 years |

**9. Do you think you should renew your first aid knowledge?**

| Yes |
| --- |
| No |
| I do not know, n. a. |

**10. IF Q 7 IS NO - Do you think you might need to acquire first aid knowledge?**

| Yes |
| --- |
| No |
| I do not know, n. a. |

**Only those, who have responded 'Yes' to the questions 8A or 8B, let answer.**

**11. What is the main reason you have not yet renewed your first aid knowledge?**

| Money (first aid course is not cheap.) |
| --- |
| Time (I have too little time.) |
| Because there is no offer for such courses. |
| Because you do not feel able to participate in the course. |
| Because i'm not interested at the moment. |
| I do not know, n. a. |

12. **Please, answer the following statement: 'Yes' if you agree, and 'No' if you disagree.**

|  | Yes | No | I do not know, n. a. |
| --- | --- | --- | --- |
| I would attend a first aid course. |  |  |  |
